# Cognitive Endpoints in Huntington's Disease Trials: A Conceptual Framework for Clinical Meaningfulness

**DOI:** 10.1002/mdc3.70298

**Published:** 2025-08-27

**Authors:** Cali M. Roiboit, Julie C. Stout

**Affiliations:** ^1^ Turner Institute for Brain and Mental Health, and the School of Psychological Sciences Monash University Clayton Victoria Australia

**Keywords:** cognitive endpoints, clinically meaningful, trials

Clinical trials conducted under regulatory conditions use prespecified outcome measures (ie, Clinical Outcome Assessments, or COAs) to establish whether a treatment has the intended effect(s).^1^ According to guidance from the United States Food and Drug Administration (FDA)^1^ COAs are required to reflect Concepts of Interest, which are signs, symptoms, or clinical events relevant to the target medical condition. For Huntington's disease (HD), Concepts of Interest include motor, cognitive, and psychiatric signs and symptoms, as well as everyday functioning.^2^ Within the clinical trial setting, Concepts of Interest vary as signs and symptoms emerge and evolve. Concepts of Interest must be observable within timeframes relevant to clinical trial durations; the idea that Concepts of Interest must align to what is relevant and observable is referred to as Context of Use.^1^ Further, the FDA highlights that COAs should indicate or predict “clinically meaningful” change, which means improvements in function, quality of life, or survival.^3^

Cognitive decline is a core HD feature across the disease span, and is well‐recognized as affecting quality of life and functioning.^4^, ^5^ Numerous cognitive measures are sensitive cross‐sectionally and longitudinally in studies of HD and are therefore prime COA candidates.^6^, ^7^ Cognitive measures set task conditions to elicit observable patient performance, producing direct, quantified evidence (eg, accuracy, timing) of a patient's capabilities, referred to as performance‐based outcomes (PerfO).^1^ For assessing cognition, PerfOs have advantages over patient‐reported outcomes (PROs) because PROs require self‐reflection related to cognitive constructs, which are inherently unfamiliar to most people or unavailable by self‐reflection (eg, executive functions, memory retrieval, selective attention, or recognition of emotions).^8^ Similar to the limitations of PROs, other observers such as family members are unable to report cognitive capabilities with sensitivity and precision.^8^

Considering their advantages, PerfOs are the FDAs preferred mode of cognitive assessment for clinical trial endpoints.^8^, ^9^ Nonetheless, the FDA questions the inherent clinical meaningfulness of cognitive PerfOs because cognitive tests that are used to elicit observable skills lack clear alignment with routine aspects of everyday life. For example, a paper‐based test of coding speed and accuracy, the Symbol Digit Modalities Test, or SDMT, remains the most widely used cognitive COA in HD. Whereas the SDMT is highly sensitive to disease severity and progression in HD,^10^ it is unlike any everyday activity. Thus, improvements or declines in performance on the SDMT alone are not sufficient for demonstrating meaningful effects that could lead to regulatory approval of a treatment. To demonstrate clinical meaningfulness using the SDMT or other cognitive tests, further evidence is required to demonstrate that beneficial changes on cognitive test performance are predictive or indicative of meaningful improvements in everyday function.^11^

Cognitive function is unarguably important as a target for new treatments across a range of neurocognitive disorders. As such, in the Alzheimer's disease (AD) drug development context, clinical trialists have been wrestling with how to establish clinically meaningful cognitive efficacy assessments for several years.^8^, ^12^, ^13^ In HD drug development, we have not yet articulated the conceptual pathways needed to bridge cognitive PerfOs to clinically meaningful outcomes. Without an agreed pathway to clinical meaningfulness in HD trials, the field remains uncertain about how to make use of the advantages presented by cognitive PerfOs within the regulatory approval pathway.

Looking to AD, early guidance recommended using co‐primary measures, aligning cotemporaneous measures of cognition with measures of daily function to establish that an improvement measured on cognition coincided with clinically meaningful benefit.^9^ Measures of daily function, however, have limited applicability in the early stages of AD when cognitive changes are detectable in advance of measurable declines in function. This timing misalignment renders the use of a co‐primary functional measures as untenable for establishing clinical meaningfulness of cognitive outcomes. In response to the conundrum posed by the requirement of clinical meaningfulness in a disease stage when functional changes are undetectable, the FDA released their 2018 guidance for AD, stating that effects on cognition alone may be considered meaningful if they are of sufficient “magnitude or breadth,” a concept that was left undefined.^11^

In the absence of specific FDA guidance, AD researchers proposed a conceptual pathway and articulated key considerations that they believed would support the claim of clinically meaningful cognitive effects.^8^, ^12^, ^13^ For example, Edgar et al^8^ created a framework in response to FDA guidance highlighting the importance of establishing the conceptual relevance of cognitive measures to everyday function and determining the magnitude of a meaningful treatment effect on cognition to establish clinically meaningful cognitive endpoints. Building on the AD work, we propose analogous frameworks to assert a path forward for claiming clinical meaningfulness of cognitive effects in HD (see Fig. 1). We also incorporate guidance from the FDA's recent Patient‐Focused Drug Development (PFDD) series, which were provided subsequently to the key AD publications.

**Figure 1:**
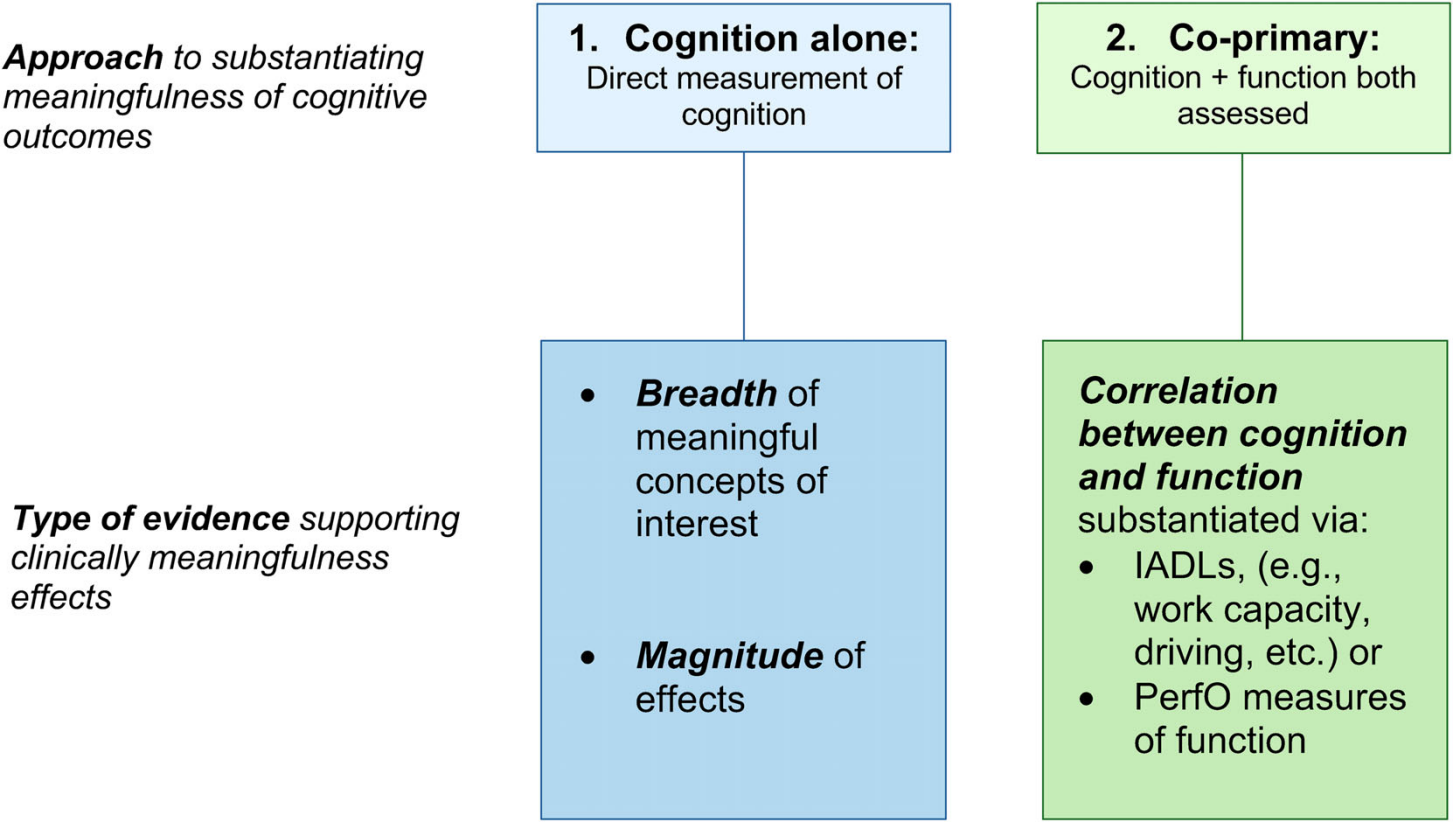
Conceptual framework for establishing clinically meaningful cognitive effects in HD clinical trials. iADL, independent activities of daily living; PerfO, performance‐based outcome measure.

## Our Framework

We propose two pathways for determining clinically meaningful cognitive endpoints HD, depending on the Context of Use of the COA: (1) determining efficacy based on cognitive effects alone (ie, using the magnitude and breadth of the effects); and (2) determining efficacy based on co‐primary measures of cognition and functions (see Fig. 1).

### Determining Efficacy on Cognitive Measures Alone

#### Conceptual Relevance

Concepts of Interest assessed by efficacy endpoints must be aligned to the understanding of the disease and must reflect the priorities of patients.^14^ The FDA's PFDD series encourages using patient‐experience data to ensure that the Concepts of Interest targeted by COAs reflect clinically meaningful outcomes.^15^ The clinical meaningfulness of particular symptoms or outcomes may vary with disease progression.^14^ Therefore, the conceptual relevance of Concepts of Interests assessed using COAs should be considered within the intended Context of Use, considering the stage of disease.^14^, ^16^ Recently, in line with the push for patient‐experience data, HD trials have supported the development and validation of novel PROs by incorporating patients into measure development,^17^, ^18^ although this approach has not yet been applied to cognitive endpoints. For cognitive measures, inclusion of patient‐experience data to determine the meaningfulness of Concepts of Interest is complicated because cognitive functions are difficult for patients to observe by self‐reflection.^19^ Nevertheless, people with HD and caregivers consistently identify cognitive decline broadly as a meaningful outcome.^4^, ^5^ Additionally, AD and Parkinson's disease studies have used qualitative methods to bolster the relevance of cognitive Concepts of Interest, setting a precedent for HD.^20^, ^21^, ^22^

The FDA's point of view is that “breadth” of cognitive effects is required to support clinical meaningfulness.^11^ Thus, the multifactorial nature of cognition means that multiple meaningful cognitive Concepts of Interest are important (eg, attention, information processing speed, memory, and executive functions). As such, cognitive endpoints should consist of batteries of tests with sufficient breadth to assess multiple complementary and meaningful cognitive outcomes. Two cognitive batteries have been most commonly applied in HD clinical trials, including the cognitive component of the Unified Huntington's Disease Rating Scale (UHDRS), which includes three tests, and the Huntington's Disease Cognitive Assessment Battery (HD‐CAB), a broader battery comprised of six tests.^23^, ^24^ While the HD‐CAB in particular demonstrates the desired breadth in assessing cognitive Concepts of Interest and the Context of Use has been demonstrated within stages of HD prior to clinical motor diagnosis and shortly after, the clinical meaningfulness of these PerfOs has not been substantiated. More research is needed to validate the relevance of the domains assessed on these measures to clinically meaningful outcomes from the patient perspective.

#### Magnitude of Effect

FDA guidance states that the magnitude of a meaningful treatment effect on COAs must be specified, which is particularly important for cognitive measures as they are indirect assessments of meaningful outcomes and thus meaningful change is difficult to interpret at face‐value.^16^ Many studies have determined what magnitudes of effects in AD endpoints signal meaningful change,^25^ although such research is limited in HD.^26^ The Minimally Clinically Important Difference (MCID) is a method preferred by regulatory bodies and commonly used with cognitive measures.^16^ MCID refers to the smallest amount of change that would be considered meaningful to patients. For neurodegenerative disorders, the magnitude of an effect that can be interpreted as meaningful differs as the disease progresses (ie, relates to the Context of Use), and as such determining the MCID for different HD stages is important to interpret change.^16^, ^25^, ^26^

MCIDs can be calculated using anchor‐ or distribution‐based methods.^16^ Anchor‐based methods involve estimating meaningful change using external measures (ideally which reflect the same Concepts of Interest) where meaningful change is already known. For example, a PRO measure assessing a patient's impression of a symptom's severity could be used as an anchor to interpret meaningful change on a multi‐item measure for the same symptom. For cognitive measures, because the clinical meaningfulness of their Concepts of Interest are yet to be demonstrated, the FDA recommends using multiple less directly related anchor measures;^16^ for example, measures of quality of life. In contrast, distribution‐based methods for determining MCID rely on statistical properties of the data (eg, standard deviation) to estimate thresholds for meaningful change.^16^ The FDA prefers anchor‐based methods because change on the COA is anchored to clinically meaningful outcomes.^16^ To date, the only study that has identified MCIDs for cognitive outcomes for clinical trials used a PRO measure of quality of life as an anchor.^26^ For example, a 1.1 reduction on the SDMT resulted in meaningful decreases in quality of life over 12‐months for people with Stage 2 HD using the Huntington's Disease Integrated Staging System (HD‐ISS).^27^ This work represents an important advancement in improving the interpretability of meaningful effects on cognitive endpoints in HD, however more research is needed to estimate MCID using various anchor measures to increase the robustness of findings.

### Determining Efficacy Using Co‐Primary Outcome Measures

#### Adapting Co‐Primary Measures for Earlier Disease Stages

For clinical trials in which cognitive decline is a treatment target, the preferred endpoint strategy is to use co‐primary measures of cognition and function.^9^, ^28^, ^29^ The co‐primary measures approach combines the sensitivity and reliability of PerfO measures of cognition with functional measures that have inherent clinical meaningfulness. There are, however, drawbacks to this approach. First, as in AD, measures of function are insensitive to the earliest signs of disease in HD when function is not yet overtly affected. For example, in HD‐ISS Stage 2, prior to clinical motor diagnosis, the dynamic range of the UHDRS Total Functional Capacity (TFC) scale, which is the most commonly used function measure in HD, is insufficient as an anchor for the precise and measurable changes that can be detected in cognitive PerfOs; all people in the HD‐ISS Stage 2 obtain perfect scores on the TFC scale despite measurable changes on cognitive tests.^27^ To address this limitation and widen the Context of Use of a functional measure, co‐primary measures for HD could incorporate more challenging, high‐level instrumental daily activities that draw more heavily on cognitive skills, which is an approach that has been useful in AD.^30^ Alternatively, PerfO measures of function could also be considered, if suitable ones can be developed and validated. Such PerfO functional measures would be both objective indicators of function and capable of assessing a broader range of readouts, such as completion times or compensatory strategy use.^31^ Evidence suggests that more challenging PerfO measures of function are sensitive in HD even in stages prior to clinical motor diagnosis (eg, measures of financial capacity),^32^, ^33^ and could complement cognitive measures after further investigation.^34^

Another important drawback of co‐primary endpoints is that larger sample sizes are required for multiple endpoints, such as the detection of effects on both cognition and function.^35^ In HD, recruiting adequate sample sizes is challenging, even when targeting participants with noticeable cognitive and functional changes as the overall population of potentially eligible people is limited, particularly in an era when multiple Sponsors are trialing potential treatments. For AD, the FDA has established a precedent for recommending a single endpoint that assesses both cognition and function: the Clinical Dementia Rating—Sum of Boxes (CDR‐SB).^30^ For HD, the UHDRS composite (cUHDRS) is a multidimensional composite of four measures encompassing cognition, function, and motor signs and the FDA has indicated that it is a suitable intermediate endpoint for HD trials.^36^, ^37^ The cUHDRS thus has promise as a clinically meaningful outcome for HD trials, however we note that for trials in which cognition is of primary interest, the cUHDRS assessments are overly limited to address the multiple significant impacts of HD on cognition.

## Final Considerations

In this paper, we borrowed from the AD literature on clinical meaningfulness of cognitive measures to outline a path forward for determining clinical meaningfulness of cognitive assessments in HD. We want to highlight, however, that AD differs from HD in an important respect. In AD, cognition has primacy as a Concept of Interest over other common symptoms that contribute to meaningful outcomes, such as changes in behavior and sleep.^38^ In HD, a broader set of Concepts of Interest converge to create disability, including cognition, motor function, and neuropsychiatric disease manifestations.^39^ Motor abnormalities have historically been given primacy as outcomes in HD clinical trials,^40^ and unlike AD, no case has been made to give primacy to cognition over other disease manifestations, or to disentangle the specific impacts of cognition versus motor versus psychiatric changes on function. Endpoint strategies with the greatest clinical meaningfulness for HD must align the broad range of cognitive, motor and neuropsychiatric COAs, the intended use of compounds being trialed, and ongoing developments in regulatory science.

## Author Roles

(1) Project: A. Conception, B. Literature Review, C. Execution. (2) Manuscript Preparation: A. Writing of the First Draft, B. Review and Critique, C. Editing.

C.M.R.: 1A, 1B, 1C, 2A, 2C.

J.C.S.: 1A, 2B, 2C.

## Disclosures

**Ethical Compliance Statement:** Ethical approval or informed patient consent was not necessary for this work. We confirm that we have read the Journal's position on issues involved in ethical publication and affirm that this work is consistent with those guidelines.

**Funding Sources and Conflict of Interest:** No specific finding was received for this work. JCS has consulted and served advisory boards for several sponsors and clinical trials over the past 20 years and is a director of a contract research organization that facilitates the implementation of cognitive assessments in HD clinical trials. JCS has been funded by the CHDI Foundation for projects aimed at developing a cognitive assessment battery for HD clinical trials. JCS provides consultation services for HD clinical trials via Stout Neuropsych Pty Ltd, and is a director of Zindametrix Pty Ltd, which facilitates the implementation of the HD‐CAB in clinical trials.

**Financial Disclosures for the Previous 12 Months:** CM;R is a recipient of the Research Training Program (RTP) scholarship funded by the Australian Government. JCS is the recipient of an Australian National Health and Medical Research Investigator grant. JCS has provided paid consulting services through Stout Neuropsych Pty Ltd to the following pharmaceutical sponsors in the past 12 months, including Skyhawk, Therapeutics, uniQure NV, LifeEdit Therapeutics, and Sage Therapeutics. JCS is also a Director of Zindametrix Pty Ltd, a contract research organization that provides services for implementation of cognitive assessments in HD clinical trials and has received compensation for Zindametrix activities in the past 12 months.

## Data Availability

Data sharing not applicable to this article as no datasets were generated or analysed during the current study.
